# Does MRI have added value in ultrasound-detected BIRADS-3 breast masses in candidates for assisted reproductive therapy?

**DOI:** 10.1016/j.ejro.2022.100474

**Published:** 2022-12-30

**Authors:** Arvin Arian, Sina Delazar, Maryam Aghasi, Behnaz Jahanbin, Nasrin Ahmadinejad

**Affiliations:** a Advanced Diagnostic and Interventional Radiology Research Center (ADIR), Imam Khomeini Hospital, Tehran University of Medical Sciences, Tehran, Islamic Republic of Iran; b Shahid Beheshti University of Medical Sciences, Tehran, Islamic Republic of Iran; c Cancer Institute, Pathology department, Imam Khomeini Hospital Complex, Tehran University of medical sciences, Tehran, Islamic Republic of Iran

**Keywords:** Breast Lesion, BIRADS, Benign Lesion, Malignant Lesion, Magnetic resonance imaging, Ultrasonography

## Abstract

**Background:**

Ultrasound-detected breast lesions with probably benign features are a great challenge for clinicians, especially in breasts with dense composition. We aimed to investigate the finding of two radiologic modalities on these lesions.

**Methods:**

This retrospective cross-sectional study recruited patients including (1) candidates of assisted reproductive therapy (ART), (2) patients with prior high-risk lesions, and (3) the “suspected” BIRADS-3 masses referring to masses that US BIRADS-3 was not compatible with the clinical breast exam. The degree of agreement in diagnosing BIRADS-3 lesions between two modalities of magnetic resonance imaging (MRI) and ultrasonography (US), and comparison of the lesions in US and MRI were the study variables.

**Results:**

A total number of 123 lesions in 67 patients with a median age of 38 (IQR: 11, range: 17–67). In the examination by MRI, 107 (87.0 %) lesions were BIRADS-3 indicating the agreement level between these two modalities. The median size of the lesions in US was 9 mm (IQR: 5, range: 3–43) and 9 mm (IQR: 10, range: 4–46) in MRI. The measured size of the lesions between the two modalities was highly correlated (Spearman correlation coefficient: 0.889, P-value < 0.001). MRI evaluation revealed two cases of deep lesions which were missed in the US imaging.

**Conclusions:**

This study found relatively high agreement values between US and MRI in detecting BIRADS-3 breast lesions in candidates for ART or patients with prior high-risk lesions. Also, MRI could downgrade about one-tenth of the cases to a lower BIRADS level and resolved the need for closer follow-up.

## 1 Introduction

Breast cancer (BC), the top cause of cancer in women, imposes a heavy burden on individuals and health systems [1], [2]. The growing incidence of BC in females made it the top rank in both sexes’ cancers in the latest updates of the GLOBOCAN 2020 global cancer estimates [1]. In this regard, appropriate screening and diagnosis approaches are needed to curb the burden of this cancer [3]. Over recent years and decades, various imaging modalities have shown to be reliable, accessible, feasible, and cost-effective in screening BC and other benign and suspected breast lesions [4], [5]. In this path, the development and expansion of newly introduced technologies like mammography and magnetic resonance imaging (MRI) have paved the way to precise diagnosis of the lesions prior to any invasive diagnostic or therapeutic procedure [6].

In order to uniformly report the imaging findings of BC screening and diagnostic assessments, unique reporting systems have been introduced and are widely used [7]. One of these extensively used reporting systems for breast lesions is the Breast Imaging-Reporting and Data System (BIRADS), which is applicable for various breast imaging modalities including ultrasonography (US), mammography, and MRI [8]. BIRADS has five reporting levels for different lesions and the third level as the focus of this study, is defined as probably benign lesions which need short-term follow-up, routinely in 6 months [9]. The BIRADS-3 diagnosed lesions are a great challenge since this diagnosis does not accurately label the lesions and needs close follow-up to reach a decision [10].

US is one of the imaging modalities which is widely accessible and easy to use to evaluate the breast lesions and has shown to be capable in the assessment of challenging lesions including BIRADS-3 ones [11], [12]. Also, breast MRI is one of the modalities with growing utilization with high sensitivity and extensive use for various lesions including BIRADS-3 [13]. BIRADS-3 lesions have various features in radiologic evaluation by US and MRI. The so-called probably benign lesions of BIRADS-3 are usually a solid mass with circumscribed margins, oval shape, moderately hypoechoic, homogenous and parallel orientation without suspicious features in US imaging [12]. On the other hand, lesions are categorized as BRADS-3 in MRI when they have oval or round shape with a circumscribed margin and homogeneous internal enhancement and plateau or persistent kinetic curve [14], [15].

Although both of these modalities are valid and widely used, variations in the findings and evaluations specifically in the case of probably benign lesions like BIRADS-3 could be challenging and confusing for radiologists and clinicians [16], [17]. In patients with dense breast composition in a primary mammography assessment, finding BIRADS-3 lesions in US imaging could be challenging and deciding for further management rely on a more precise diagnosis. In this regard, we aimed to present MRI features of US detected BIRADS-3 lesions and compare the MRI and US findings among patients with ultrasound detected BIRADS-3 masses who also performed MRI imaging by clinician request.

## 2 Material and methods

### 2.1 Study design and population

We designed a retrospective cross-sectional study to investigate the aim of this manuscript. The study took place at one of the referral hospitals in Tehran, Iran. The patients were recruited for the study from January 2019 to September 2021. Data were explored retrospectively and no intervention was done on the included patients. The inclusion criteria were classified in three groups: [1] candidates of assisted reproductive therapy (ART), [2] patients with prior high-risk lesions (their lesions may already have been removed or planned to have follow-up according to histopathologic results), and [3] the “suspected” BIRADS-3 masses referring to masses that US BIRADS-3 was not compatible with the clinical breast exam. Exclusion criteria were lack of US or MRI imaging data and other relevant demographic and clinical information.

### 2.2 Imaging protocols

An expert radiologist with 15 years of experience in breast imaging did the US examination on patients with a high-resolution US device to detect any suspected lesions. Breast MRIs was done with a standard protocol including axial 3D T1-weighted nonfat suppressed, axial T2-weighted with fat suppression, DWI (b-values of 50, 400 & 1000 s/mm^2^), axial 3D T1 weighted fat suppressed pre- and post-contrast dynamic series every 60 s up to 360 s, and finally sagittal and coronal reformat on subtracted T1-weighted images using a 3-Tesla MRI device (GE discovery system).

### 2.3 Patient follow-up

Patient follow-up was done for the included sample based on each individual and their diagnosis. BIRADS-2 lesions in MRI were considered benign so they needed no follow-up. BIRADS-3 lesions were followed up to at least one year and they were stable. BIRADS-4 lesions underwent core needle biopsy and finally were resected due to their size.

### 2.4 Study variables

Patients’ age, personal history, and family history of BC were the demographic variables reported in this study. US and MRI BIRADS category (which was assigned according to American College of Radiology (ACR) guideline 2013) and the measured size of the lesions were the main study variables. According to the ACR guideline for breast cancer screening by MRI, criteria for BRADS-3 lesions in MRI are oval or round shape with a circumscribed margin and homogeneous internal enhancement and plateau or persistent kinetic curve [14], [15]. Also, the additional features of the lesions in the MRI investigation described in this study were background parenchymal enhancement (BPE), type, enhancement, and curve of the lesion.

### 2.5 Statistical analysis

The descriptive presentation of the results included the median and interquartile range (IQR) of the quantitative variables and the frequency and percentage of the qualitative variables. Spearman correlation coefficient was used to examine the bivariate correlation of two quantitative variables. P-values less than 0.05 were assumed statistically significant. All statistical analyses were conducted by the SPSS Statistics 16 software (Released 2007. SPSS for Windows, Version 16.0. Chicago, SPSS Inc.).

## 3 Results

### 3.1 General findings

A total number of 123 lesions in 67 patients with a median age of 38 (IQR: 11, range: 17–67) were included in this study. A minority of patients reported a family history of BC including 6 (8.9 %) with history in first-degree relatives, 7 (10.4 %) in second-degree relatives, and 2 (3.0 %) in third-degree relatives. Also, only 5 (7.5 %) patients had a prior personal history of BC in this study.

### 3.2 Radiologic evaluation

All evaluated lesions in the US examination were BIRADS-3. In the examination by MRI, 107 (87.0 %) lesions were BIRADS-3 indicating the agreement level between these two modalities. Also, the other levels in the MRI investigation included 12 (9.8 %) lesions as BIRADS-2, and four (3.3%) lesions as BIRADS-4 (Table 1). Among the BIRADS-2 lesions, six cases were intramammary lymph node, one complicated cyst, one bloody cyst, one galactocele, two cysts, and one granulomatous mastitis fluid collection in MRI. Also, regarding the BIRADS-4 lesions, three cases were finally diagnosed with fibroadenoma and one with phyllodes tumor Table 2 summarizes the characteristics of patients with discordant findings of the suspected lesions in US and MRI assessment.

**Table 1:** Statistics of lesions with discordant findings in US and MRI examinations.

	US	MRI			Agreement Rate (%)
	BIRADS-3	BIRADS-2	BIRADS-3	BIRADS-4	
Number of lesions (%)	123 (100 %)	12 (9.8 %)	107 (87.0 %)	4 (3.3 %)	87

US: Ultrasonography, MRI: Magnetic Resonance Imaging.

**Table 2:** Further details of the discordant lesions found in MRI examination.

MRI	BIRADS	Number	Final diagnosis
	2	6	Intramammary lymph node
		2	Cyst
		1	Complicated cyst
		1	Bloody cyst
		1	Galactocele
		1	Granulomatous mastitis with fluid collection
	4	3	Fibroadenoma
		1	Phyllodes tumor

MRI: Magnetic Resonance Imaging.

The median size of the lesions in US was 9 mm (IQR: 5, range: 3–43). The median size of the lesions in MRI was also 9 mm (IQR: 10, range: 4–46). The measured size of the lesions between the two modalities was highly correlated (Spearman correlation coefficient: 0.889, P-value < 0.001).

The additional features of the lesions in MRI were as follows. The BPE was mild in 28 (22.8 %) lesions, minimal in 18 (14.6 %) lesions, moderate in 8 (6.5 %) lesions, marked in 1 (0.8 %) lesion, and also asymmetric in 1 (0.8 %) lesion. The type of lesion was mass in 81 (65.9 %) lesions, cyst in 11 (8.9 %) lesions, and focus in 3 (2.4 %) lesions. The enhancement of lesions was positive in the majority (66.7 %) of the evaluated lesions. Also, the curve was persistent in a majority (65.0 %) of the detected lesion in the MRI.

## 4 Discussion

The main findings of this study were the relatively high agreement between US and MRI modalities in the detection of the BIRADS-3 breast lesions and the highly significant correlation between the size of the lesions in two imaging.

In this study, we tried to describe the findings of two imaging modalities and their agreement on the BIRADS-3 breast lesions. The so-called “probably benign” breast lesions classified as the third level of the BIRADS may be challenging in diagnosis and have indefinite imaging and clinical features [18]. A systematic review and meta-analysis study on the BIRADS-3 lesions found that malignancy rates in these lesions varied between 0.5 % and 10.1 % among studies. The highest rates were in non-mass lesions, mass lesions, and foci [19]. Another study showed that among the three mentioned subtypes of this classification, the masses have the highest probability of turning to malignancy in the long-term follow-up [13]. Therefore, added knowledge of these challenging lesions through timely screening, follow-up, and proper therapeutic interventions is necessary to prevent adverse events like malignancy.

In the current investigation, we included the detected lesions as BIRADS-3 in the US examination, and breast MRI did the second level of investigation. Various approaches and algorithms could be utilized to use these modalities in evaluating the breast lesion; however, the best approach is to use them per case and based on the characteristics of each individual [20]. Further improvements in the breast US have shown to improve the detection of breast lesions; for example, a study showed that the combination of the conventional US and the contrast-enhanced US had better diagnostic performance and had higher agreement rates with the breast MRI findings in differentiating benign and malignant breast lesions [21]. Another study showed that contrast-enhanced US is a reliable and accessible method for evaluating breast lesions as it could provide typical enhancement patterns of the lesions similar to what could be found in the breast MRI examination [22].

The prevalence of hereditary BC and those with a high risk of the malignancy due to a family history of BC was not high in our included sample in this study and we could not draw a pattern to describe the efficacy and precision of the US and MRI in the detection of the BIRADS-3 lesions. The literature shows that breast MRI is superior to US and mammography for screening and diagnosing lesions in women with hereditary BC [23]. In this regard, MRI could detect the lesions in the high-risk population with higher sensitivity and in earlier stages [24]. In the case of challenging populations and lesions like the BIRADS-3, the results of different modalities vary among individuals and trials are going on to find out the best approach [25].

**Fig. 1:**
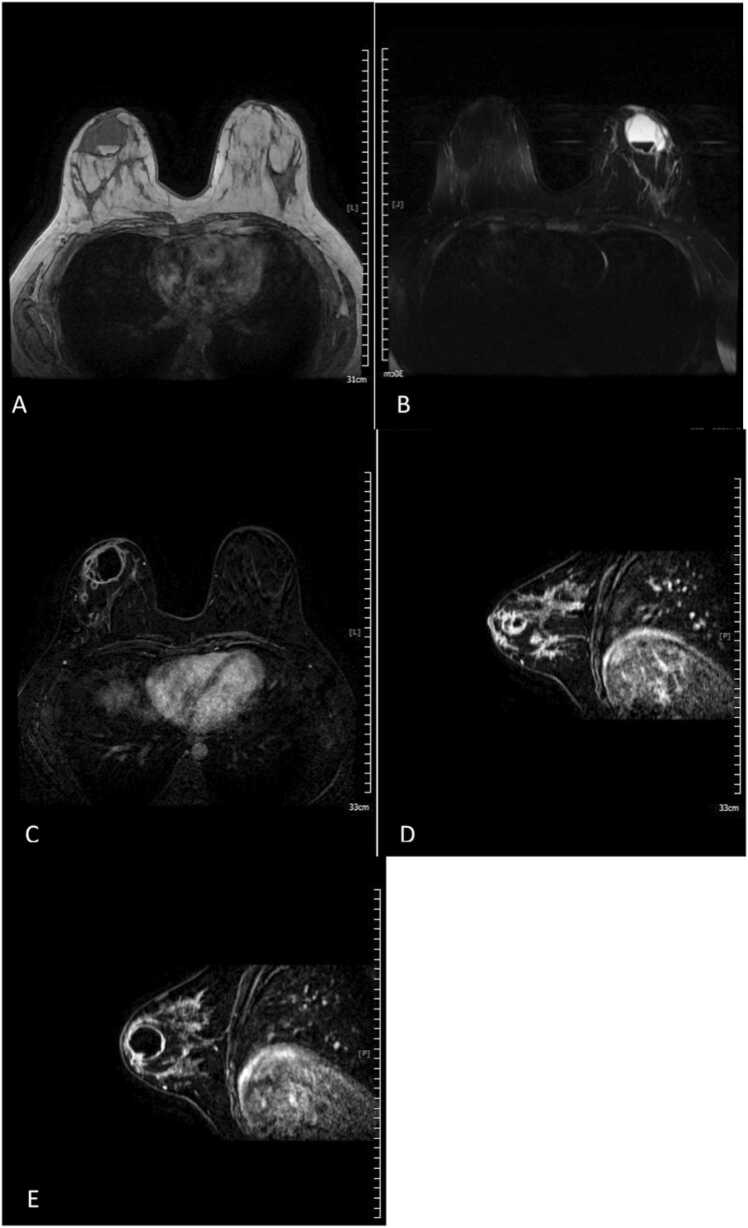
Axial T1-weighted (A), Axial T2-weighted (B), and Axial and sagittal post contrast T1 (C–E). Images show a thick rim enhancing blood fluid cystic mass in right breast retro areole region along with multiple smaller cysts in surrounding parenchyma.

**Fig. 2:**
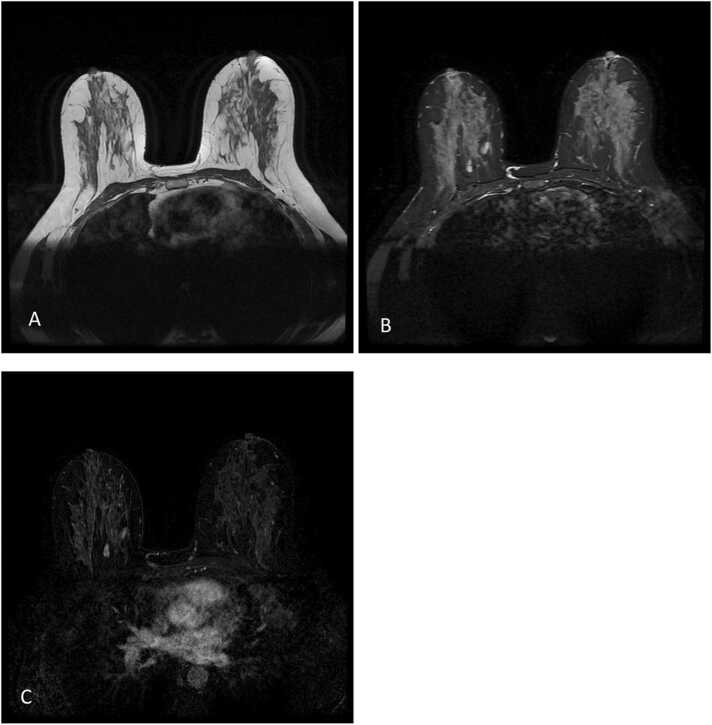
Axial T1-weighted (A), Axial T2-weighted (B) and Axial post contrast T1-weighted (C). Images show circumscribed persistent enhancing mass in right breast deep central zone.

**Fig. 3:**
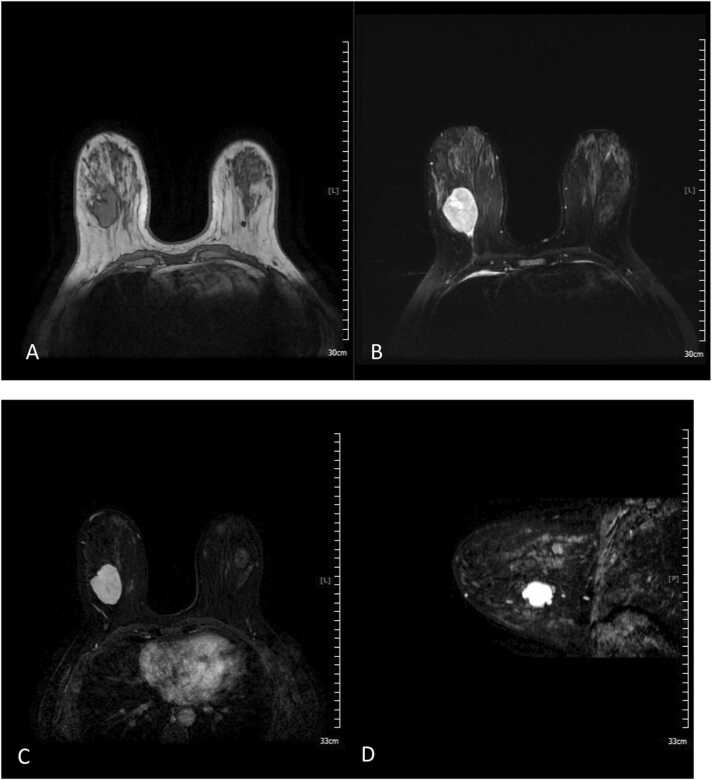
Axial T1weighted (A), Axial T2 weighted (B), Axial and sagittal post contrast T1 (C and D). Images show a hyper enhancing mass with micro lobulated margin measuring 45 * 30 mm in right breast LOQ region with surgical excision pathology result of phyllodes.

**Fig. 4:**
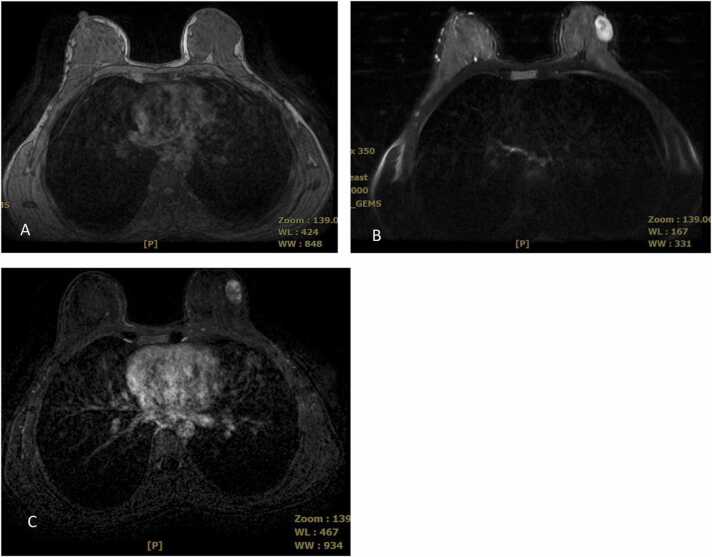
Axial T1-weighted (A), Axial T2-weighted (B) and Axial post contrast T1-weighted (C). Images show circumscribed heterogeneously enhancing mass in left breast UOQ with surgical excision pathology result of fibroadenoma.

**Fig. 5:**
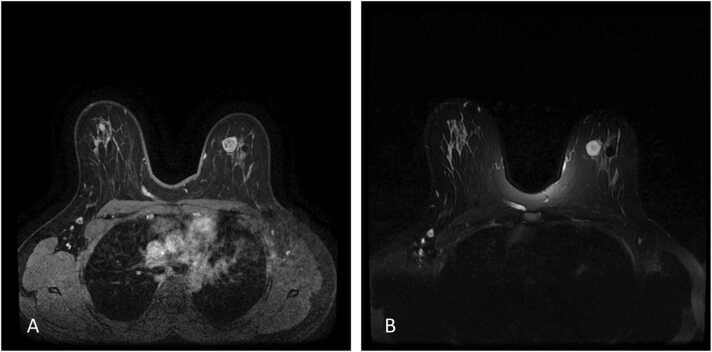
Axial T2-weighted (A) and axial post contrast image (B) demonstrate a circumscribed round mass with high signal intensity on T2-weighted and heterogenous enhancement with surgical excision pathology result of fibroadenoma.

**Fig. 6:**
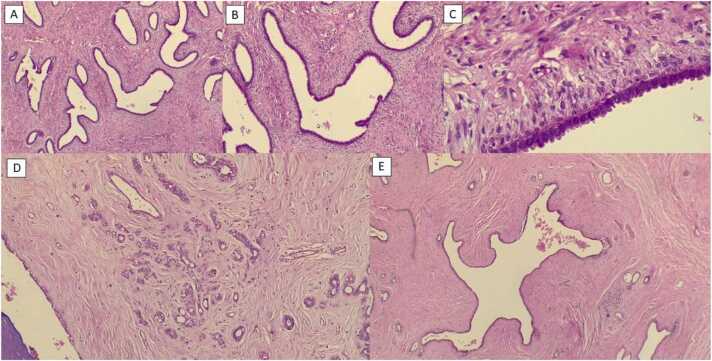
Examples of pathologic samples assessment from the included patients. (A) A fibroepithelial lesion with stromal hypercellularity and prominent leaf-like pattern (40 × magnification), (B) presence of mild stromal cellularity with periductal accentuation without atypia or increased mitosis (100 × magnification), (C) Benign phyllodes tumor shows peri ductal accentuation (400 × magnification), (D) fibroadenoma with focus of sclerosing adenosis, (E) a cyst with 4 mm diameter (100 × magnification).

The current study found some discordance in suspected lesions between the US and MRI examinations. We believe the detected discordance could be due to many factors, as explained as follows. In US examination, cystic lesions were assumed as complicated lesions due to having internal echo and internal septa; however, in MRI, the cysts were seen as a fluid collection without enhancement and therefore were downgraded to BIRADS-2 lesions. Also, circumscribed hypoechoic masses suggesting probably benign lesions in US were assigned BIRADS-3 level; however, in MRI the high T1 cysts without enhancement were assumed as bloody cysts or high proteinaceous lesions suggesting BIRADS-2 level. One case of a dilated duct with circumscribed intraductal mass lacking vascularity and vascular pedicle in US was found and was assigned as a BIRADS-3 lesion; however, it was upgraded to a level 4 lesion in MRI due to detection of a cyst with a thick wall and heterogeneous enhancement (Fig. 1). Finally, we should consider that lesions with BIRADS-3 characteristics in MRI and without such findings in US, could be due to the deep location of some of these masses (Fig. 2). Also, if the later mentioned lesions were not circumscribed in MRI or had heterogeneous enhancement patterns in MRI, they were upgraded to BIRADS-4 lesions (Fig. 3, Fig. 4, Fig. 5). Also, some examples of the pathologic assessment of the lesions obtained from patients are presented in Fig. 6.

This study had some limitations. The limited sample size was the main limitation that needed to be stated. Expanding the study for more significant sample sizes to include more diverse patterns and pathologies of BIRADS-3 breast lesions is suggested for future research in this field. Also, incorporating other imaging modalities like mammography is highly suggested.

## 5 Conclusions

This study described and analyzed findings of MRI assessments of patients with suspected BIRADS-3 lesions in US imaging who were candidates for ART and also the cases with prior history of high-risk breast lesions. The findings showed that the two modalities have high rates of agreement on these lesions and both could help to differentiate these challenging lesions. As an important finding, MRI utilization in this study could downgrade about one-tenth of the cases to a lower BIRADS level and resolved the need for closer follow-up and this adds to the knowledge in the field. Although the MRI evaluation in this study could reveal a few cases with deep lesions which were missed in the US imaging, the wide use of full protocol MRI for evaluation of suspected BIRADS-3 lesion remains a controversy and needs further studies. Also, besides the full protocol MRI, the abbreviated MRI method could also be used in these patients to rule out higher BIRADS lesions and this might be an idea for future studies.

## CRediT authorship contribution statement

**Arvin Arian:** Conceptualization, Methodology, Writing – original draft, Writing – review & editing. **Sina Delazar:** Conceptualization, Methodology, Writing – original draft, Formal analysis, Data curation Visualization, Writing – review & editing. **Maryam Aghasi:** Formal analysis, Investigation, Data curation, Writing – review & editing. **Behnaz Jahanbin:** Investigation, Data curation, Visualization, Writing – review & editing. **Nasrin Ahmadinejad:** Conceptualization, Methodology Writing – review & editing, Supervision.

## Funding statement

This study received no grants or funding support.

## Declaration of Interest statement

The authors declare no conflicts of interest.

## Data Availability

The datasets generated and analyzed during the current study are not publicly available due institutional requirements, but are available from the corresponding author on reasonable request.
